# Topics of Public Interest

**Published:** 1907-10

**Authors:** 


### TOPICS OF PUBLIC INTEREST.

---

### The Committee on the Prevention of Tuberculosis of The Charity Organization Society of the City of New York

CONSUMPTION CATECHISM FOR CHILDREN.

A consumption catechism for school children is the subject of a pamphlet being printed by the Department of Health of the city of New York for distribution in the schools of the city. Through the help which has been promised by the department of education it is expected to get this catechism into the hands of every one of the 600,000 and more children attending the public schools. Another large group of children will be secured it is expected from parochial and private schools. As these cards will bear the imprint "Take this card home and show it to your family and friends," and as it is planned to have the teachers give this same advice to their pupils, this will prove the most widespread and thorough distribution yet attempted in this country of printed instructions on the subject of consumption.

In a series of thirty-two questions and answers the catechism briefly and simply tells what consumption is, how it is conveyed from person to person, "how you can keep from getting it," "how you can keep others from giving it to you" and how it is cured. Added to the catechism is a list of the associated special tuberculosis dispensaries and a map of the city showing the district allotted to each one of these.

Although the pamphlet is primarily designed for school children it contains much material which will be of help to their parents and older brothers. Such an answer as that given to the question "What are the first signs of the disease?" will warn many an unsuspecting person that an examination by a competent physician should not be put off. "Loss of strength, cough, fever in the afternoon and loss of weight, sometimes bleeding or hemorrhage of the lungs and the coughing up of sputum or phlegm are the first signs that the unwary are now told to look for. After describing how one person infects another through the germs which are contained in the spit of the consumptive or in the invisible droplets sprayed out when consumptives cough or sneeze it is stated that those who are sickly or rundown from disease, overwork or intemperance and whose systems cannot fight the bacilli are those most likely to get consumption, just as the ordinary cold or cough if neglected is the most common sickness that develops into consumption. Thorough cleaning and disinfection of houses or rooms newly moved into are urged as one essential safeguard against the consumption germs which a careless consumptive may have left in rooms occupied by him.

"Even if the tubercle bacilli get into the lungs of a healthy person they are usually killed there" it is stated, and so the lesson is plain that the first great rule to keep from getting consumption is simply "keep as well as possible." To do this four things are recommended, fresh air, proper food, cleanliness and temperance in all things. If a cough lasts more than two weeks an examination of the lungs by a competent doctor or at a special tuberculosis dispensary is advised. A minimum program for cleanliness is set forth in two warm baths a week and in cleaning house with damp brooms and cloths, while for air it is stated that every study and living room should be aired several times a day and one window in the bed room kept full half open all night.

The catechism in answer to the question "Is it dangerous to live or work with a consumptive?" answers "no, not if he is careful and clean; careful to destroy all the sputum he coughs up and never to spit on the floor or streets." It is said that consumption can be cured if treatment is begun early by good food, fresh air and rest and such medicines as the doctor may prescribe. If a consumptive cannot go to a country sanatorium he is advised to go to a doctor or a dispensary, to keep out in the fresh air and sunlight as much as possible, to keep his windows open day and night and not to waste time or money on patent medicines or advertised cures.

---

### New York State Historical Association

ADDRESS OF HON. ANDREW S. DRAPER, STATE COMMISSIONER OF EDUCATION—GREAT STATE OF NEW YORK—LET NOT HER CHILDREN FORGET HISTORIC PAST, SAYS COMMISSIONER DRAPER—NEW ERA BEGINNING—OUR DESCENDANTS WILL WONDER WHY WE FOUND THE PRESENT PROBLEMS DIFFICULT—SEVERAL OTHERS READ PAPERS—OFFICERS CHOSEN.

The ninth annual meeting of the New York State Historical Association was held at the Buffalo Historical Building September 17, and 18, 1907. Dr. Andrew S. Draper addressed the association Tuesday evening, September 17. He spoke on New York's Obligations to Her History.

Judge Draper's address attracted a large audience, many prominent educators being present to hear the commissioner speak on this interesting subject who applauded him loudly and long.

It is plain that Dr. Draper is an optimist, and not a pessimist. While he deplores that so comparatively little has been done with the history of so notable a state, he has no fears for the future. Dr. Draper contends that New York is near the center of everything historic in American annals. Even the first blood of the Revolution was shed in New York and not in Boston, he said.

During his address, Dr. Draper said:

"We must lay no claim to what is not justly ours. But we owe it to our fathers and to our children to prevent literary fiction and much repetition from perverting a true understanding of historic facts. The first blood of the Revolution was spilled in New York and not in Boston. Every home in our sparsely settled state was in deadly danger from the capture of Ticonderoga till the Indians were driven from the Hudson river to the Genesee country.

"What memories the names of Cobleskill, Schoharie, Cherry Valley, Springfield, Schenectady, Unadilla, Canajoharie, German Flats, Minisink and many others may well revive! Oriskany in severity and in results was a heavier battle than Bunker Hill, and Saratoga was the decisive engagement of the war. Of course, all that was done in New York was not done by New York men, but with main reliance upon volunteer soldiers the men of this state were necessarily at the forefront of any warfare within its borders. Forced by their situation to bear the leading part in the Revolution, the men of New York, as Wayne wrote Washington in the middle of the night after the capture of Stony Point, behaved like men determined to be free.

"Our fathers were not much given to leaving records for their children. Those children have been frequently unmindful, often indifferent, sometimes inaccurate. We have uniformly been engrossed with innumerable activities and the very volume of our history makes it difficult to popularise it. Many have come among us in later years who are valiantly helping us to make more history, who can not easily appreciate our early history, for they are a part of the story of another people struggling for the democracy which has come to be more stable here than in any other land under the sun. The writers of American history have, for the most part, lived in other states and they have written under the spell which other associations impose upon them. We have not been much aided by song and story; at times we have been injured by literary humor which other people seem unable to grasp. The children in the schools, often the students in the colleges and universities, more often still the men and women of our busy cities and towns, know but little of the splendid story and appreciate all too lightly the obligations which it imposes.

"But, after all, the writing of history is not the only way of expressing our obligations to the makers of it. Here we are, 8,000,000 of every kind of people that the sun ever shone upon, proving the stability and the potentiality of a pure democracy. We are not in peril; we confide absolutely in our security. Discussion is freer, sentiment makes more rapidly and conclusions are surer and sounder than ever before. We can do anything we think well to do. The commercial primacy of the state seems sure enough, but endless measures are in progress to make it doubly sure. The national centers of the publishing business, of finance and of manufactures are within our borders. The problem of absolute democracy in religion has been worked out to a complete solution. So the great problem of democracy in education is well advanced and the solution is inevitable.

"We are now in the midst of complete applications of the fundamental principles of our democracy to our industries. We are beginning to make and to enforce laws which will promote all the just interests of both capital and labor and limit the improper exercise of the organised power of each. Our children will wonder that we had so much trouble making it clear that the common power can only be used in the common interest, and that in our business as well as in our religion, our education, and our politics, every child of the nation is to have his free and equal chance. If we make it completely so, as seems likely enough, we shall show to all the world that democracy opens opportunity to moral and material progress and we shall discharge a part of the obligation which our generation of freemen owes to the generations of freemen who have gone before."

The society elected the following named officers: President, James A. Roberts; first vice-president, Grenville M. Ingalsbe; second vice-president, Sherman Williams; treasurer, James A. Holden; secretary, Robert O. Bascom; assistant secretary, Charles F. King.

At Tuesday afternoon's meeting papers were read by F. H. Severance, George D. Emerson and Major Louis L. Babcock of Buffalo; Jacques W. Redway of Mount Vernon, N. Y., and Professor George K. Hawkins of Plattsburg. Peter A. Porter read a paper at Wednesday's session. Mr. Emerson's paper was a review of the career of General Winfield Scott, particularly the soldier's part in the Niagara Frontier campaign of 1812.

---

### The Amended Agricultural Law

(*New York Tribune*, September 4, 1907.)

WORK FOR MILK USERS—MUST CLEAN BOTTLES—NEW LAW REQUIRES CONSUMER TO AID—EFFORTS FOR PURITY.

Any householder or consumer of milk who fails to clean properly the can or other receptacle in which the milk was delivered is liable to prosecution under the recently amended agricultural law of this state. This applies not only to the residents of this city but to those of all the cities of the state. Inspectors of the State Department of Agriculture are empowered by this statute to seize such cans or bottles and use them as evidence.

The law relates alike to the dealer and user, and it presumes that any milk shipped within a city is for purposes of consumption, such shipping being prima facie evidence of that intention. All particles must be cleaned from the can or bottle by rinsing with water or in some other way. The delivery to any railroad for shipping of an uncleaned empty milk can is prohibited. Every use, return, delivery or shipment of an unclean can shall be deemed a separate violation of the law.

Cream and curd are included with milk within the meaning of this act, and the word "curd" used in this connection applies to pot cheese or cottage cheese.

Handlers of milk in this city say that a small percentage of the cans and bottles is cleaned by the consumer after being emptied. Less than half, it is said, are rinsed in water, and only a small percentage of this number is cleaned in any other way. Frequently these uncleaned bottles are allowed to remain in a box for days at a time, and then it is almost impossible for the dealers to get them clean. The milk and cream particles cling to the sides and top, and the result is that frequently fresh milk or cream is emptied into the bottles, which are sealed and come back to the city with the milk contaminated.

The law prohibits the placing of "sweepings, refuse, dirt, litter, garbage, filth or any other animal or vegetable substance liable to decay and tending to promote or produce an unsanitary condition" in milk cans. There are fewer violations of this paragraph than in the case of the bottles, but it is not entirely obeyed.

Several of the large wholesale firms in the city, it was reported yesterday, had informed their customers that cans must be cleaned before their return. It was thought probable that such action might be taken in the case of bottle customers unless a marked change in the condition of the bottles collected is shown soon.

H. H. Kracke, the Assistant Commissioner of Agriculture in charge of the work of that department in this city, said that the inspection of milk cans by his men is proceeding satisfactorily at the ferries, and that a great improvement in the condition of the cans is found. The other night at Barclay street twelve thousand cans were inspected, and not one was set aside as failing to comply with the law regarding cleanliness.

In the case of the bottles the commissioner and his men found a different condition, a large percentage of the latter showing that they had been returned to the dealers uncleaned and that the old milk particles had not been removed through whatever cleaning they received later. It was explained that it is almost impossible to remove these if they are permitted to stand for twenty-four hours.

Local dealers who were looking for an increase in the demands of the farmers when the time for making October contracts come, regard with considerable alarm the action of the dealers in Orange County in increasing the price of milk sold in the villages there from five to six cents a quart. The supply is short, and the farmers who have been selling milk at a loss for some time have come to a point where they do not feel like longer continuing.

Now that they have considered it necessary to raise the price at their own home, it is not expected, it was said here yesterday, that these same men, who send a large amount of milk to New York, would continue doing so at a loss. Contracts for the six months beginning October 1 will be signed within a week or ten days, and practically every wholesaler is prepared to listen to demands for more money.

The question of selling milk by dry or liquid measure has been brought up by a number of farmers in connection with the agitation for higher prices. Some appear to think that they are not getting a fair deal when their milk is bought by dry measure. The manager of one of the largest milk houses told a *Tribune* reporter yesterday that for several years most of the milk in New York has been sold by dry measure—practically by the pound. In explanation of this custom it was said that after milk cans have been used a short time they are dented and will not hold forty quarts as before they fell into the hands of the baggage men.

To avoid paying for the dents, wholesalers sometime ago hit upon the idea of buying milk by weight. A price which was said yesterday to be perfectly fair to both the producer and the consumer was agreed upon, and transactions have been conducted on that basis. It is a purely arbitrary matter, no formal action having been taken at any time by either the dealers or the farmers.

The quality of the milk that is delivered here is good, but it was said yesterday that there was no increase in the quantity, and famine possibilities have not disappeared.

---

Aneurysm of the Arch of the Aorta.—George L. Peabody presents the history and autopsy of an obscure case of aneurysm of the descending arch of the aorta that was diagnosed only by means of an *x*-ray photograph a few days before rupture into the esophagus. There were many symptoms of hysteria and no history of syphilis or other etiological factor of aneurysm.—*Medical Record,* April 13, 1907.

